# Resveratrol may reverse the effects of long-term occupational exposure to electromagnetic fields on workers of a power plant

**DOI:** 10.18632/oncotarget.17668

**Published:** 2017-05-07

**Authors:** Dan Zhang, Yang Zhang, Baoyu Zhu, He Zhang, Ye Sun, Chengxun Sun

**Affiliations:** ^1^ Electrical Power Research Institute, Jilin Electrical Power Company Limited, Changchun 130021, Jilin, China

**Keywords:** ELF-EMFs, high-voltage electricity lines, resveratrol, inflammatory, biomarkers of oxidative stress

## Abstract

High-voltage electricity lines are known to generate extremely low-frequency electromagnetic fields (ELF-EMFs). With the process of urbanization, increasing concerns has been focused on the potentially hazardous impacts of ELF-EMF on human health, and the conclusions are controversial. Little is known about the method of prevention against ELF-EMF induced healthy problems. A total of 186 male workers with occupational exposure to high-voltage electricity lines, and 154 male subjects with insignificant exposure as reference control were enrolled in this study. Resveratrol or placebo was given as dietary supplements (500 mg twice daily), and several inflammatory biomarkers and biomarkers of oxidative stress were assessed. Workers who had long-term exposure to high-voltage electricity lines exhibited elevated urinary levels of 8-hydroxy-2-deoxy-guanosine (8-OHdG) and F2-isoprostane, compared to the reference group. Lower plasma nuclear factor kappa B (NF-κB) and interleukin (IL)-6 were observed in exposed workers compared to the reference group. Resveratrol significantly reversed the adverse impacts of ELF-EMF. Stimulated cytokine production by resveratrol was found in exposed workers but not in the reference group. This study supported that occupational and long-term exposure to high-voltage electricity lines has an adverse effect on homeostasis of human body, and resveratrol supplement could be an effective protection strategy against the adverse effects induced by ELF-EMFs.

## INTRODUCTION

High-voltage electricity lines are known to generate extremely low-frequency electromagnetic fields (ELF-EMFs). Since 1970, a number of researches have investigated the potentially hazardous impacts of ELF-EMFs on human body but the conclusions remain controversial [1]. With the increasing process of urbanization, electric power plants are extensively established, which raised concerns from the public on the potentially hazardous effects of ELF-EMFs on workers and nearby residents. International Agency for Research on Cancer (IARC) has classified ELF-EMFs as ‘possible carcinogenic to humans’ in 2002, because previous results suggested an association between ELF-EMFs and childhood leukemia [2].

A study on human osteosarcoma MG-63 cells indicated that ELF-EMF may induce cell apoptosis through impairing redox homeostasis and activating the p38 mitogen-activated protein kinase (MAPK) [3]. Increase of reactive oxygen species (ROS) by ELF-EMFs was observed in another *in vitro* study, and it was correlated with enhanced neurotoxin 1-methyl-4-phenylpyridinium (MPP) in Parkinson's disease [4]. Evidence showed that long-term exposure to ELF-EMFs elevated the incidence of chronic myeloid leukemia in female mice [5]. On the contrary, a recent study using human cancer cell lines demonstrated that long-term exposure of ELF-EMF could repress cancer cell growth while increase mitochondrial activities [6]. The possibilities of ELF-EMF application on clinical therapy have been proposed by several studies, where ELF-EMF could accelerate wound healing *via* inducing anti-inflammatory state, increasing endothelial cell proliferation and stimulating collagen formation [7]. The inverse consequences of ELF-EMFs may be dependent on its amplitude, as suggested in a previous investigation which demonstrated that ELF-EMF could repress angiogenesis when used in a specific window of amplitude [8].

Epidemiological investigations showed that ELF-EMFs was a possible risk factor for acute myeloid leukemia and follicular lymphoma among workers with occupational exposure [9]. ELF-EMFs have also been reported to have an adverse effect on the brain, resulting in neurodegenerative diseases and brain tumor [1]. However, many evidences were inconsistent, and no association of ELF-EMFs with the susceptibility to human cancer has been observed [10]. Therefore, to date, the effects of ELF-EMFs on human health remain controversial.

In this study, we aimed to analyze the effects of occupational ELF-EMF exposure on several inflammatory biomarkers and biomarkers of oxidative stress. Additionally, *ex vivo* stimulation of interleukin (IL)-6, IL-8, IL-1β and tumor necrosis factor-alpha (TNF-α) was performed. Previous studies have demonstrated the biochemical and physiological functions of resveratrol, including protective effects against cardiac oxidative stress and alleviation of inflammation [11, 12]. As a natural polyphenol found in a variety of plants, its clinical applications in diseases prevention are promising. Therefore, we assessed whether daily intake of resveratrol supplements alters the influence of ELF-EMF on the health of exposed workers.

## RESULTS

**Figure 1:**
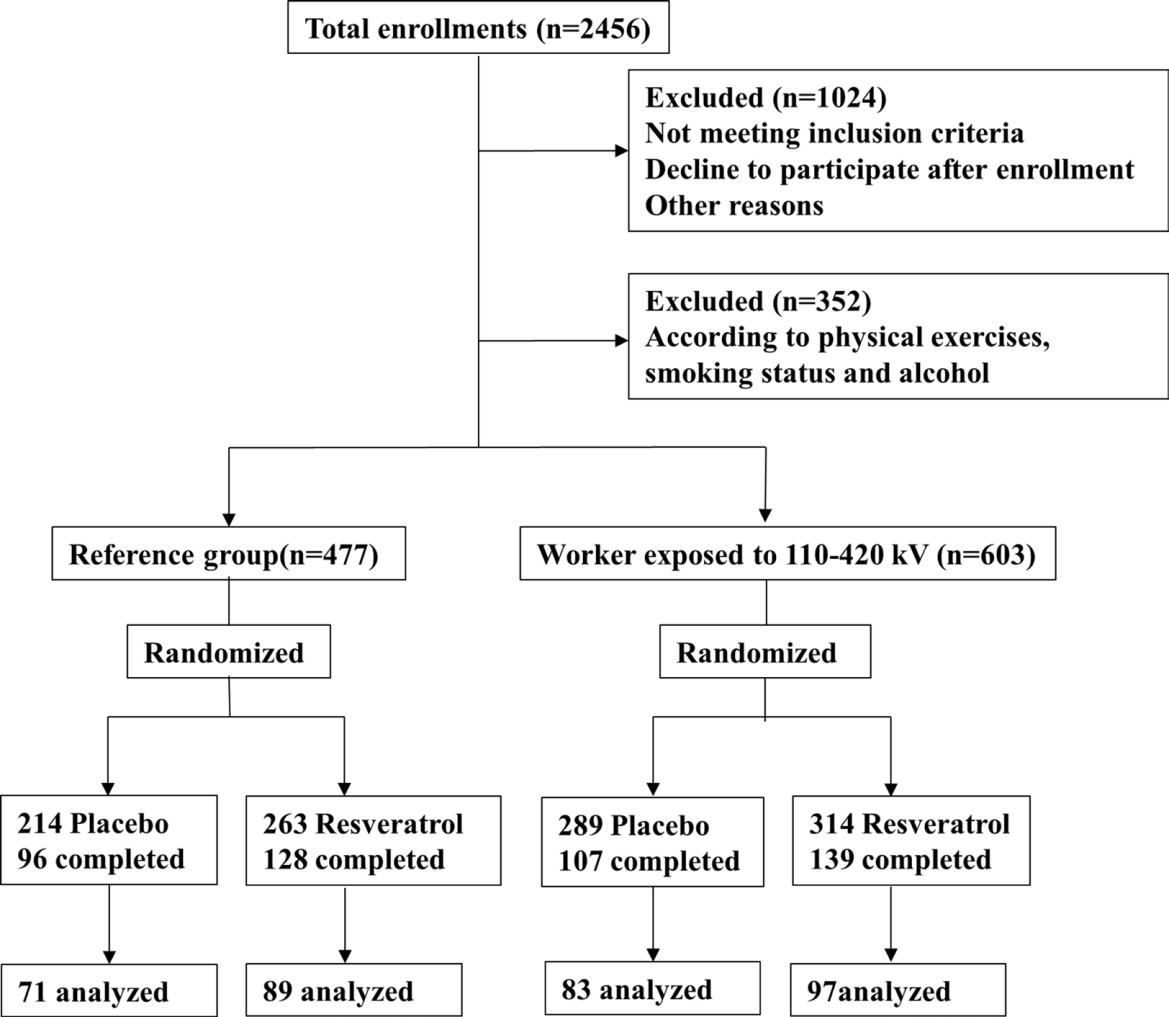
Flow diagram of subjects screening A total of 2456 subjects were enrolled in the current study. Workers exposed under 110–420 kV and the reference participants were treated with placebo or resveratrol.

To eliminate the influence of variants on the outcomes of this study, we selected the participants with similar age and BMI ranges. After inclusion and exclusion (Table 1), the remaining 1080 participants were then divided into two groups according to their ELF-EMF exposure (Figure 1). Among these 1080 participants, 603 were occupational workers with long-term exposure and the rest 477 had no significant exposure. There were 610 dropouts in the study due to various reasons: those who did not follow the study protocol that 500 mg resveratrol needs to be taken twice daily, and who did not attend the whole assessments. A few of the participants were diagnosed of mild illness, such as cold, cough and pharyngitis, and refused to continue the protocol or was not able to attend the assessments. The rest of the drop-out participants were excluded because they did not fill in the questionnaire.

**Table 1:** Inclusion and exclusion criteria

Inclusion criteria
Workers exposed to high-power lines for more than 20 years
Reference groups were at same age range as exposed groups
Insignificantly exposed to electric field for reference groups
Male
Participants were living far from high-voltage power lines.
Willing to give informed consent
Willing to have dietary supplementation intake and underwent blood tests
Exclusive criteria
History of regular intake of green tea, vitamin C and vitamin E in the past year
Inheritance disorders in family
Body mass index (BMI) < 18.5 or > 30 kg/m^2^
history long-term exposure to high-voltage power lines

### Characteristics of the participants

Table 2 illustrated the general characteristics of the 340 eligible participants who completed the study. Since body temperature may be elevated by chronic inflammation, it was also tested in the two groups and no significant difference was observed. However, we found that workers with long-term exposure to ELF-EMFs showed significantly higher incidence of chronic diseases, such as high blood pressure and cardiovascular complications, as well as health complaints including headache, pain and depression, compared to the reference group.

**Table 2:** Characteristics of subjects with no or long-term occupational exposure to extremely low electromagnetic field emitted from high voltage power lines

	Reference group (*N* = 89)	>20 years exposure (*N* = 97)	*P*
Age, years	565±2	554 ± 6	0.162
BMI, kg/m^2^	243 ± 1	242 ± 7	0.425
Body temperature	36 ± 41 ± 7	36 ± 62 ± 3	0.299
Self-reported physical exercise, hours	96 ± 1	95 ± 7	0.865
Occupational exposure, h/day	-	61 ± 2	-
Mobile phone usage (hr/week)	31 ± 8	31 ± 2	0.223
Computer usage (hr/week)	204 ± 6	213 ± 9	0.624
Chronic diseases in 5 years (%)	20 ± 1%	23 ± 4%*	0.021
Health complaints (pain, headache, depression) (%)	24 ± 5%	30 ± 7%*	0.012

Data are presented as mean ± SD, *p value < 0.05.

### The outcomes of resveratrol supplementation

Compared to placebo (Supplementary Tables 1 and 2), daily dietary intake of resveratrol was found to significantly reverse the adverse effects of ELF-EMFs on exposed workers. Urinary 8-OHdG and F2-isoprostane levels were significantly elevated in workers with long-term exposure to high-voltage electricity lines, compared with the reference group (Table 3), and were repressed after 12-months of resveratrol supplements. No significant differences in serum melatonin and HSP70 were found between the exposed worker group and the reference group. Plasma NF-κB and IL-6 levels were down-regulated in exposed workers compared to those in reference group (Table 4). These observations suggested that resveratrol significantly reversed the impacts of ELF-EMFs on human body.

**Table 3:** Levels of biomarkers of oxidative stress in workers exposed to high-voltage power lines and control group before and after resveratrol supplementation

	Reference group (*N* = 89)	> 20 years exposure (*N* = 97)	F	*P*
Before resveratrol supplementation				
Biomarkers of oxidative stress				
8-OHdG (mg/mmol creatinine)	25 ± 310.2	29 ± 87.5**	11.881	0.01
F2-isoprostane (ng/mg creatinine)	14 ± 77.7	20 ± 46.4***	30.332	< 0.001
Serum melatonin (pg/ml)	19 ± 68.5	20 ± 10.5	0.181	0.671
Serum HSP70 (ng/L)	1.4 ± 0.5	1.5 ± 0.5	1.857	0.175
After 12-month of resveratrol supplementation				
Biomarkers of oxidative stress
8-OHdG (mg/mmol creatinine)	24.6 ± 9.9	28.0 ± 4.4**#	9.421	0.002
F2-isoprostane (ng/mg creatinine)	15.2 ± 8.3	18.4 ± 7.2**#	7.923	0.005
Serum melatonin (pg/ml)	19.0 ± 11.8	19.6 ± 9.8	0.144	0.706
Serum HSP70 (ng/L)	1.2 ± 0.8	1.2 ± 0.7	-	NS

Data are presented as mean ± SD. *, ** or *** represent the comparison between exposed workers and reference group while # or ## represents the comparison between the individuals before and after supplementation. * and ^#^*p* value < 0.05; ** and ^##^*p* value < 0.01; ***p value < 0.001; NS, non-significant.

8-OHdG, 8-hydroxy-2-deoxy-guanosine.

NF-κB, nuclear factor kappa B.

TNF-α, tumour necrosis factor-alpha.

HRC, high-sensitive C-reactive protein.

IL, interleukin.

**Table 4:** Plasma levels of inflammatory biomarkers and *ex vivo* stimulation of cytokine production in workers exposed to high-voltage power lines and control group before and after resveratrol supplementation

	Reference group (*N* = 89)	> 20 years exposure (*N* = 97)	F	*P*
Before resveratrol supplementation				
Inflammatory biomarkers in plasma				
NF-κB, ng/L	1038 ± 60.2	1020 ± 57.5*	4.35	0.038
HRP mg/L	0.7 ± 0.7	0. 7 ± 0.9	-	NS
IL-6 (pg/mL)	1.1 ± 1.0	0.8 ± 0.6*	6.271	0.013
*Ex vivo* stimulation of cytokine production				
TNF-α ng/ml	56.3 ± 9.2	52.7 ± 11.1*	5.744	0.018
IL-6 ng/ml	72.2 ± 2.5	63.8 ± 23.3**	6.991	0.009
IL-1β ng/ml	33.1 ± 1.2	29.2 ± 2.6*	5.201	0.024
IL-8 ng/ml	4.4 ± 2.3	4.1 ± 2.6	0.679	0.407
After 12-month of resveratrol supplementation				
Inflammatory biomarkers in plasma				
NF-κB, ng/L	1043 ± 55.2	1041 ± 54.5#	0.061	0.804
HRP mg/L	0.7 ± 1.2	0. 76 ± 1.2	0.119	0.734
IL-6 (pg/mL)	1.0 ± 0.8	1.11.5##	0.314	0.576
*Ex vivo* stimulation of cytokine production				
TNF-α ng/ml	57.5 ± 14.9	55.7 ± 17.1#	0.581	0.447
IL-6 ng/ml	69.8 ± 19.8	67.5 ± 21.6##	0.570	0.451
IL-1β ng/ml	35.1 ± 16.4	33.9 ± 12.6##	0.316	0.575
IL-8 ng/ml	4.2 ± 2.0	4.3 ± 2.2#	0.105	0.747

Data are presented as mean ± SD. * or ** represent the comparison between exposed workers and reference group while # or ## represents the comparison between the individuals before and after supplementation. * and ^#^*p* value < 0.05; ** and ^##^*p* value < 0.01; NS, non-significant.

8-OHdG, 8-hydroxy-2-deoxy-guanosine.

NF-κB, nuclear factor kappa B.

TNF-α, tumour necrosis factor-alpha.

HRC, high-sensitive C-reactive protein.

IL, interleukin.

### Cytokine production

The abilities of cytokine production in all participants were measured using *ex vivo* stimulated production of IL-6, IL-8, IL-1β and TNF-α by LPS (Table 3). We found that exposed workers exhibited reduced cytokine production after LPS stimulation, as lower levels of these cytokines were observed in exposed worker group than those in the reference group. However, 12-month of resveratrol significantly rescued the reductions, whereas no obvious change was found in the placebo group (Supplementary Table 2).

## DISCUSSION

With the increasing electric power demands, artificial EMF emitted from power transmission lines and/or power plants has raised concerns about their potentially hazardous effects on human health. However, the association between ELF-EMFs and human health are controversial due to inconsistent data reported. Occupational exposure to ELF-EMFs may be a potential danger for workers of power plant. In this study, we employed 1080 subjects including workers in a power plant. All subjects were supplied with dietary resveratrol for 1 year and we observed significant differences in the oxidative stress and immune responses between exposed workers and reference group.

In this study, both urinary 8-OHdG and F2-isoprostane levels were elevated in workers exposed to high-voltage electricity lines compared to the reference group. In the last decade, 8-OHdG has been frequently used as a biomarker for oxidative DNA damage because it is the final product of guanine oxidation. The presence of 8-OHdG in the DNA may give rise to an accumulation to gene mutations and tumorigenesis because of its molecular instability. Evidence showed a correlation between elevated 8-OHdG and carcinogenesis in various tumors [13, 14]. Urinary F2-Isoprostane also serves as a sensitive indicator of oxidative stress, and an increasing number of studies consistently demonstrated associations between F2-isoprostane and cancers [15, 16]. Thus, elevations of urinary 8-OHdG and F2-isoprostane in exposed workers suggest that long-term ELF-EMF increases oxidative stress in those workers and may develop cancer eventually. Consistently, a number of previous studies have demonstrated that oxidative stress induced by ELF-EMFs may lead to DNA damage [17] in a dose-dependent way [19]. These results were contradicted by several *in vitro* studies, showing an absence of DNA damage in cells exposed to 60-Hz electromagnetic field [20]. However, the results from meta-analysis and clinical studies support our findings that ELF-EMF increases oxidative stress in workers [21]. Interestingly, a recent study reported a lower level of DNA damage in welders who were occupationally exposed to ELF magnetic fields, compared with the unexposed group [23]. It was speculated that exposure to chromium, nickel and/or other metals may lead to the different results between theirs and other investigations. A tendency of DNA damage induced by ELF-EMFs could potentially result in mutagenesis [24]. The subjects in this study were occupationally exposed to ELF-EMF for more than 20 years, therefore the elevated levels of urinary 8-OHdG and F2-isoprostane observed may be a cumulative effect. It is difficult to conclude whether these elevations result in DNA damage and mutagenesis or not, but nevertheless these potential risk factors cannot be ignored.

In recent years, melatonin and HSP70 have been shown to exert essential functions in anti-tumor immunity [25, 26]. Although it was reported earlier that ELF-EMFs significantly upregulated *Hsp70* gene expression [27], ELF-EMFs did not change the plasma level of HSP70 protein in this study. Early studies demonstrated that ELF-EMF may suppress the synthesis of melatonin [28]. We also found no statistically significant difference in plasma melatonin between the two groups. It can be implied that the effect of ELF-EMF on oxidative may be not mediated by repressing these defenses against oxidation stress in cells.

In the present study, long-term exposure to occupational ELF-EMFs was shown to result in significantly lower levels of inflammatory biomarkers including NF-κB and IL-6, suggesting that ELF-EMFs may affect the immune system to reduce inflammatory response, in which NF-κB pathway could be involved. A previous epidemiological study also reported that plasma NF-κB was lower in workers with EMF exposure [29]. Few studies have focused on the influence of ELF-EMFs on NF-κB signaling. A study found that ELF-EMFs increased active form of NF-κB in cells [17]. NF-κB is a crucial factor in a number of cellular processes, such as cell proliferation, apoptosis and immune responses. A variety of inflammatory stimuli can induce the active form of NF-κB to transfer from cytoplasm into the nucleus, where it stimulates the transcriptional activities of target gene such as cytokines. It can be implied that long-term exposure to ELF-EMFs may repress NF-κB signaling, which in turn may suppress inflammatory processes. Dysregulation of NF-κB was reported to be implicated in several diseases including B-cell malignancies and diabetes mellitus [30]. Under normal circumstances, NF-κB exerts protective functions in cells against oxidative stress [31]. Thus, ELF-EMF may block this crosstalk of oxidative stress and NF-κB signaling, which may elicit damages of organelle and cellular functions. Nevertheless, we are unable to conclude that the reduced plasma NF-κB level benefits human health, because impaired NF-κB activation may also result in abnormal T or B cell responses.

Our results revealed a decreasing trend of plasma IL-6 in exposed workers compared to it in reference group. In addition, *ex vivo* stimulated productions of TNF-α, IL-1β, IL-6 and IL-8 by LPS were performed, which in part indicates the cytokine production ability of the monocytes. Interestingly, the productions of TNF-α, IL-6, IL-1β and IL-8 after LPS stimulation were significantly lower in exposed worker group than those in the reference group. Both plasma concentrations and *ex vivo* stimulated productions of cytokines in exposed workers were significantly elevated to the concentrations in reference group after 1 year of resveratrol supplementation. Thus, resveratrol significantly attenuated the influences of ELF-EMF on cytokine production. In 2012, Carta et al. reported that oxidative stress sign in stressed monocytes (high ROS and damaged mitochondria) was worsened by TLR stimulation and therefore inhibit protein synthesis, which is responsible for the impaired production IL-6 [32]. It is possible that resveratrol suppresses oxidative stress and repairs damaged mitochondria induced by ELF-EMF to reverse the ability of cytokine production from monocytes. In another recent study, they found increases in serum LDL after EMF exposure [33]. Lipids are important moleculars involved in maintaining the functions of mitochondria. Elevated level of LDL in serum may suggest an abnormal status of mitochondria. However, further evidence is needed to demonstrate the status of mitochondria after long-term exposure to ELF-EMF. Earlier studies reported that pulsed electromagnetic fields repressed pro-inflammatory cytokine secretion including TNF-α, IL-6, IL-1β and IL-8 [34, 35]. Although repression of cytokines has been reported as an effective approach to inhibit inflammatory process, deficiency in cytokine production from monocytes after LPS stimulation may also lead to an abnormal immune response. In this study, if we combine these results with the observations that higher rates of chronic diseases and health complaints were found in workers with occupational exposure to high-voltage electricity lines, the reductions in plasma NF-κB level and cytokine production in the whole blood may imply the adverse effects of ELF-EMFs on human health.

For subjects randomly selected from the populations, the rates of chronic hypertension and cardiovascular complications and pain were supposed to be similar in exposed and non-exposed groups. However, we found that the rates of chronic diseases and health complaints (pain, headache, depression) in exposed workers were significantly higher that reference group. There may be an association between the health status and ELF-EMF-induced oxidative stress. Oxidative stress plays critical role in several chronic complications including hypertension, cardiovascular complications, dyslipidemia and depression [36–38]. Commonly, oxidative stress stimulates inflammation response by interacting with NF-κB signaling and cytokines secreted from cells with activated NF-κB signaling may protect cells by down-regulating oxidative stress. However, as shown in our results, low levels of several pro-inflammatory cytokines were observed in workers after long-term exposure to ELF-EMF, indicating that the aberrant inhibition may lead to further accumulation of oxidative stress. Thus, the higher incidence of chronic diseases and health complaints may be associated with elevated oxidative stress induced by ELF-EMFs. Further studies are necessary to clarify the effects of dietary resveratrol on those chronic complications.

## MATERIALS AND METHODS

### Participants

Participants were recruited for the present study in Jilin First People's Hospital between September 2010 and August 2014. The Institutional Ethic Board of Jilin First People's Hospital has approved this study. Recruitment was advertised in a power plant in Jilin, China. In addition, we also invited the participants to advertise this project to their families and friends. Electrical line workers who are commonly exposed to 110–420 kV electricity lines were chosen as the experimental group. The main tasks of these electrical line workers are installing, repairing, and maintaining the high-voltage electricity lines. EFA-300 meter was used to measure the occupational EMF intensity with frequency ranging from 5 Hz to 32 kHz, and was placed under the high-voltage electricity lines. The electric range of the instrument is 0.1 V/m–316 kV/m, and the magnetic field range is 0.1 nT–32 mT, both of which have an accuracy of ± 3%. We found that the average intensity of EMF reached 1426 V/m for electric fields and 20 μT for magnetic fields. In order to ensure there was no significant exposure in the control group, we recruited families or friends of the workers in the power plant, and none of them was significantly exposed to high voltage electricity lines.

All of the participants were confirmed to be healthy after physical examination, as well as blood and urine tests before enrollment. Doctors and trained nurses were sent to the power plant to collect blood and urine samples. Since the subjects in the reference group were not workers in the power plant, they went to the power plant on the day of sample collection. Informed consents were signed by all participants.

### Exclusion and inclusion criteria

The average time of occupational exposure to 110–420 kV was calculated according to the self-reported durations of daily occupational exposure. Enrolled participants were divided into two separate groups: 1) workers exposed to 110–420 kV for more than 20 years; 2) reference group without significant exposure. The characteristics of the two groups were recorded and compared. Inclusion and exclusion criteria of the current study were according to both scientific and ethical considerations, as illustrated in Figure 1. We selected the subjects of ages ranging from 40 to 60 years and BMI ranging from 21 to 30 kg/m^2^. Additionally, participants were excluded if they were smoker, alcoholic, or residentially exposed to high-voltage powerlines during their life time. All of these participants were Chinese, thus, the results of this study may only be applicable in Chinese population. None of the participants were exposed to hazardous chemicals and participants with previous electric shocks were excluded.

### Resveratrol supplementation

For each group, the participants were randomized into two subgroups treated with either placebo or resveratrol. Resveratrol were supplied as dietary supplements to each participant (500 mg twice daily) for a period of 12 months. It was demonstrated that 28 days of resveratrol administration (2.5 or 5 g once daily) mg significantly decreased fasting and postprandial glucose and insulin in patients with type 2 diabetes [39]. Kennedy in 2010 found that resveratrol (250 mg or 500 mg) increased cerebral blood flow compared to placebo and suggested a dose-dependent action of resveratrol for improving cerebral blood flow [40]. However, a previous study reported mild to moderate gastrointestinal symptoms caused by the 2.5 and 5 g doses of resveratrol [41]. Therefore, to avoid side effects and maximum the efficiency of resveratrol, we chose 500 mg twice daily as the dose of resveratrol administration. In our previous pilot study, we treated subjects with 2 months of resveratrol supplementation but none significant results have been observed. A previous clinical study showed that 12 months of a grape extract supplementation containing resveratrol significantly reduced the expression levels of several pro-inflammatory cytokines including IL-1β and TNF-α [42]. Thus, in this study, we investigated the effects of resveratrol on subjects with one year dietary intake.

At the end of study, 470 of them have completed the entire study protocol, whereas 610 were dropped out. After questionnaire, participants who had more than 20 hours self-reported physical exercise and comparative long computer and/or mobile phone usage time per week were excluded from participating in the following protocols in this study.

### Body mass index (BMI) measurements

Body height and weight of all participants were taken twice by trained staff of the hospital, who were blind to the group assignments. Body weight was measured with participants in light clothing and without shoes using a digital weighing scale at an accuracy of 0.1 kg. Body height was measured as a standing posture without shoes at an accuracy of 0.1 cm.

### Questionnaire on health history

An in-depth health history questionnaire, consisting of questions concerning information on demographics, hypertension, diabetes, alcohol intake and use of medication including vitamin supplementation, were issued to all enrolled participants. Participants who did not complete the questionnaire or were on regular vitamin supplementation were excluded from the study. According to previous studies, vitamins such as vitamin C and vitamin E and green tea may affect oxidative damage and total antioxidant capacity. However, because of the involvement of oxidative stress measurements in this study we excluded participants who have regular intakes of vitamin supplements and green tea to eliminate their influences.

### Biomarkers measures

Urine samples were collected from all participants at months 0 and 12 of resveratrol supplementation. For all participant, urine samples in 24 h were collected into plastic sample cups pre-filled with 3 g ascorbic acid, which were maintained at 4°C and were collected during the visit on the next day. We purified urine samples by mixing with 3× ethanol (in volume). All mixtures were cooled to 4°C, and then centrifuged for 20 min at 1500 × *g*. The supernatant was discarded, and the pellet was placed in a speed vacuum to vaporize the residual ethanol. All blood samples were collected under fasting conditions early in the morning at months 0 and 12 of resveratrol supplementation. Serum was obtained after centrifugation at 1,200 × *g* for 10 min at 4°C and stored at –80°C until further analysis. F2-isoprostanes were analyzed in the purified samples in triplicate *via* a competitive ELISA kit (Cayman Chemical, MI). The detection limit of this assay was 3 pg/ml. F2-isoprostane concentrations were normalized with creatinine concentration (Cayman Chemical). Urinary 8-hydroxy-2-deoxy-guanosine (8-OHdG) was determined by the 8-OHdG EIA competitive assay Kit purchased from Cayman Chemical. The assay has a detection range from 10.3–3,000 pg/ml and a sensitivity of approximately 30 pg/ml. The level was normalized with creatinine (Cayman Chemical). Serum melatonin level was measured by a commercial ELISA kit (Immuno Biological Laboratories, Hamburg GmbH, Hamburg), which has a detection range 3.0–350 pg/ml, with intra- and inter-assay coefficients of variation (CV) 6.4% and 9.1%, respectively. Serum human heat shock protein (HSP70) was analyzed by HSP70 assay kit purchased from Bio Medical Assay (BMASSAY, Beijing) following manufacturer's manuals. The detection limits of this assay was < 0.05 ng/mL, and the intra- and inter assay CVs of HSP70 were 6.5% and 8.2% respectively. Nuclear factor kappa B (NF-κB) was determined by an ELISA kit (R&D Systems, MN) following manufacturer's manuals, and the intra- and inter-assay CVs were 5.7% and 8.2%, respectively. Serum high-sensitivity C-reactive protein (HRP) was assessed using an ELISA assay (Cayman Chemical). The assay range was 46.9–3,000 pg/mL, and thentra- and inter-assay CVs were 5.1% and 4.7%, respectively. Serum IL-6 concentration was determined using an ELISA kit (R&D Systems), according to the manufacturer's instructions. The assay standard range was from 0.156 to 10 pg/ml, and the intra- and inter-assay CVs were 7.6% and 9.1%, respectively.

### *Ex vivo* stimulated production of IL-6, IL-8, IL-1β and TNF-α by lipopolysaccharide (LPS)

*Ex vivo* stimulated productions of IL-6, IL-8, IL-1β and TNF-α were measured as previous reported [24] with citrate-treated whole blood. Lipopolysaccharide (LPS, Sigma, MO, USA) was added into whole blood within 60 min of collection to a final concentration of 2.5 μg/mL using sterile polypropylene tubes. Control group was parallel to experimental group without LPS stimulation. The samples were first incubated for 24 h at 37°C, after which the tubes were centrifuged at 1,000 × *g* for 10 min. Supernatants were then frozen at −80°C until further cytokine measurements. Multiplex bead kits (Biosource, Camarillo, CA, USA) were employed to test the stimulated levels of IL-1β, IL-6, IL-8 and TNF-α, with both inter- and intra-assay CVs less than 10%.

### Statistical analysis

All data were presented as mean ± standard deviation (SD). The Kolmogorov–Smirnov test or analysis of variance (ANOVA) was applied for continuous variables. The nonparametric Mann-Whitney *U* test was employed for comparing abnormal distributed variables. Two or more ways ANOVA analysis were used to compare the differences in biomarker levels between exposed and reference groups. A student's paired *t-test* was used to compare the levels of each biomarker before and after resveratrol. SPSS/PC+™ software (Version 20, IBM Co) was used for statistical analyses. Descriptive data is presented as mean ± standard deviation (SD). Differences were considered statistically significant at *P* < 0.05.

## CONCLUSIONS

In summary, the current study found that long-term occupational exposure to high-voltage electricity lines has an adverse effect on oxidative stress and immune response among workers. High oxidative stress and DNA damage risk have been observed in exposed workers compared to those in the reference group. Additionally, immune responses are comparably different in exposed workers from reference group. However, resveratrol significantly reversed the impacts of long-term exposure to ELF-EMF in workers. This study supported that occupational and long-term exposure to high-voltage electricity lines may play an adverse effect on human health, and resveratrol supplement could be effective as a protection strategy against ELF-EMFs. Our result only investigated prolonged and occupational exposure of ELF-EMF so that the results may not be applied for short term or other types of exposures to ELF-EMF.

## SUPPLEMENTARY MATERIALS TABLES


