# Supplementary material for: Resveratrol may reverse the effects of long-term occupational exposure to electromagnetic fields on workers of a power plant
Source: Oncotarget. 2017 May 7;8(29):47497–506. doi: 10.18632/oncotarget.17668 (PMC5564581; doi:10.18632/oncotarget.17668)
Supplement: Supplementary file 1 [file oncotarget-08-47497-s001.pdf]

Resveratrol may reverse the effects of long-term occupational exposure to electromagnetic fields on workers of a power plant

Supplementary Materials

Supplementary Table 1: Levels of biomarkers of oxidative stress in workers exposed to high-voltage power lines and control group before and after placebo

	Reference group (*N* = 71)	> 20 years exposure (*N* = 83)	F	*P*
Before resveratrol supplementation				
Biomarkers of oxidative stress				
8-OHdG (mg/mmol creatinine)	24.9 ± 9.6	27.9 ± 7.1*	5.936	0.016
F2-isoprostane (ng/mg creatinine)	16.3 ± 6.8	19.4 ± 6.2**	10.577	0.001
Serum melatonin (pg/ml)	19.6 ± 7.3	20.5 ± 6.9	0.747	0.389
Serum HSP70 (ng/L)	1.4 ± 0.7	1.5 ± 0.4	1.46	0.228
After 12-month of placebo				
Biomarkers of oxidative stress				
8-OHdG (mg/mmol creatinine)	25.7 ± 11.5	30.5 ± 10.3**	9.017	0.003
F2-isoprostane (ng/mg creatinine)	15.1 ± 9.0	19.4 ± 7.6**	12.460	0.001
Serum melatonin (pg/ml)	20.1 ± 8.0	19.9 ± 9.1	0.021	0.886
Serum HSP70 (ng/L)	1.3 ± 0.8	1.3 ± 0.7	-	NS

Data are presented as mean ± SD. * or ** represent the comparison between exposed workers and reference group. *p value < 0.05; **p value < 0.01, NS, non-significant.

8-OHdG, 8-hydroxy-2-deoxy-guanosine.

NF-κB, nuclear factor kappa B.

TNF-α, tumour necrosis factor-alpha.

HRC, high-sensitive C-reactive protein.

IL, interleukin.

Supplementary Table 2: Plasma levels of inflammatory biomarkers and *ex vivo* stimulation of cytokine production in workers exposed to high-voltage power lines and control group before and after placebo

	Reference group (*N* = 71)	> 20 years exposure (*N* = 83)	F	*P*
Before resveratrol supplementation				
Inflammatory biomarkers in plasma				
NF-κB, ng/L	1039 ± 47.2	1025 ± 43.5*	4.432	0.037
HRP mg/L	0.7 ± 0.4	0. 7 ± 0.5	-	NS
IL-6 (pg/mL)	1.2 ± 0.9	0.9 ± 0.6*	5.791	0.017
Ex vivo stimulation of cytokine production				
TNF-α ng/ml	55.2 ± 9.1	52.5 ± 9.2*	4.039	0.046
IL-6 ng/ml	74.7 ± 9.4	64.7 ± 22.1**	10.674	0.001
IL-1β ng/ml	34.1 ± 0.1	31.3 ± 9.9*	4.765	0.03
IL-8 ng/ml	4.3 ± 2.1	4.5 ± 2.6	0.329	0.567
After 12-month of placebo				
Inflammatory biomarkers in plasma				
NF-κB, ng/L	1033 ± 40.4	1018 ± 48.9*	4.217	0.042
HRP mg/L	0.7 ± 1.0	0. 6 ± 1.3	0.279	0.598
IL-6 (pg/mL)	1.1 ± 1.0	0.8 ± 0.7*	4.751	0.031
Ex vivo stimulation of cytokine production				
TNF-α ng/ml	55.7 ± 10.2	52.1 ± 10.1*	4.522	0.035
IL-6 ng/ml	67.0 ± 10.7	63.4 ± 9.2*	5.041	0.026
IL-1β ng/ml	37.2 ± 6.0	34.7 ± 6.2*	6.409	0.012
IL-8 ng/ml	4.5 ± 1.9	4.0 ± 1.7	2.970	0.087

Data are presented as mean ± SD. * or ** represent the comparison between exposed workers and reference group. *p value < 0.05; **p value < 0.01.

8-OHdG, 8-hydroxy-2-deoxy-guanosine.

NF-κB, nuclear factor kappa B.

TNF-α, tumour necrosis factor-alpha.

HRC, high-sensitive C-reactive protein.

IL, interleukin.
